# Structural Analysis of a Novel Cyclohexylamine Oxidase from *Brevibacterium oxydans* IH-35A

**DOI:** 10.1371/journal.pone.0060072

**Published:** 2013-03-26

**Authors:** I. Ahmad Mirza, David L. Burk, Bing Xiong, Hiroaki Iwaki, Yoshie Hasegawa, Stephan Grosse, Peter C. K. Lau, Albert M. Berghuis

**Affiliations:** 1 Department of Biochemistry and Groupe de Recherche Axé sur la Structure des Protéines, McGill University, Montreal, Quebec, Canada; 2 State Key Laboratory of Drug Research, Shanghai Institute of Materia Medica, Chinese Academy of Sciences, Zhangjiang Hi-Tech Park, Pudong, Shanghai, China; 3 Department of Life Science & Biotechnology and ORDIST, Kansai University, Suita, Osaka, Japan; 4 National Research Council Canada, Montreal, Quebec, Canada; 5 McGill University, Departments of Microbiology and Immunology and Chemistry, Montreal, Quebec, Canada; 6 FQRNT Centre in Green Chemistry and Catalysis, Montreal, Quebec; Instituto de Tecnologica Química e Biológica, UNL, Portugal

## Abstract

Cyclohexylamine oxidase (CHAO) is a flavoprotein first described in *Brevibacterium oxydans* strain IH-35A that carries out the initial step of the degradation of the industrial chemical cyclohexylamine to cyclohexanone. We have cloned and expressed in *Escherichia coli* the CHAO-encoding gene (*chaA*) from *B. oxydans*, purified CHAO and determined the structures of both the holoenzyme form of the enzyme and a product complex with cyclohexanone. CHAO is a 50 kDa monomer with a PHBH fold topology. It belongs to the flavin monooxygenase family of enzymes and exhibits high substrate specificity for alicyclic amines and *sec*-alkylamines. The overall structure is similar to that of other members of the flavin monooxygenase family, but lacks either of the C- or N-terminal extensions observed in these enzymes. Active site features of the flavin monooxygenase family are conserved in CHAO, including the characteristic aromatic cage. Differences in the orientations of residues of the CHAO aromatic cage result in a substrate-binding site that is more open than those of its structural relatives. Since CHAO has a buried hydrophobic active site with no obvious route for substrates and products, a random acceleration molecular dynamics simulation has been used to identify a potential egress route. The path identified includes an intermediate cavity and requires transient conformation changes in a shielding loop and a residue at the border of the substrate-binding cavity. These results provide a foundation for further studies with CHAO aimed at identifying features determining substrate specificity and for developing the biocatalytic potential of this enzyme.

## Introduction

Amine oxidations are observed in a wide range of biological processes, including the metabolism of polyamines and neurotransmitters in higher organisms and as a source of ammonium in lower eukaryotes and bacteria. The oxidation of biological amines to their corresponding imines is catalyzed by two distinct groups of proteins: the quinoprotein class of enzymes and the flavin-containing amine oxidases [1], [2]. The flavin-containing amine oxidases carry out the oxidative cleavage of the α-CH bond of the amine to form an imine product while reducing a flavin cofactor. This unstable compound then undergoes aqueous hydrolysis to yield the corresponding aldehyde and either ammonia or an amine, depending on the substrate. In the final step of the catalytic cycle, the reduced flavin reacts with molecular oxygen to yield hydrogen peroxide and regenerate the oxidized flavin cofactor [3].

The monoamine oxidases (MAOs) are flavin-containing amine oxidases that are widely distributed in higher eukaryotes where they oxidize the primary amino groups of arylalkyl amine substrates [4]. There has been considerable pharmaceutical interest in these enzymes as they are involved in the metabolism of catecholamine neurotransmitters such as dopamine, noradrenaline and serotonin. Inhibition of these enzymes raises the levels of monoamine neurotransmitters in the brain, making them attractive targets for the therapy of neurological disorders such as Parkinson’s disease. The crystal structure of human monoamine oxidase B (MAO B) was the first three-dimensional atomic structure determined of a MAO, providing the first glimpse into the structure-function relationship in this class of enzymes [5]. The structure of MAO B is compact and globular, with a three-domain topology consisting of cofactor-binding, substrate-binding and transmembrane binding regions. The topology of the substrate- and cofactor-binding domains is similar to that initially described in *p*-hydroxybenzoate hydroxylase and is found in several other proteins with flavin cofactors. Subsequent structures of human and rat monoamine oxidase A (MAO A) have added to our understanding of these enzymes in mammalian systems [6], [7].

In contrast to the well-characterized mammalian MAOs, relatively little is known about the structure of these enzymes in the lower eukaryotes and prokaryotes [8]–[10]. To date, there is only one three-dimensional structure of a MAO from the fungus *Aspergillus niger* (MAO N) and none from a bacterial source [11]. The MAO from *A. niger* has shown considerable promise as a biocatalyst in the deracemization of chiral amines, materials with pharmaceutical and agrochemical applications whose synthesis remains challenging. Directed evolution experiments with this enzyme have yielded mutants that exhibit higher catalytic turnover and improved activity towards chiral secondary amines [12], [13]. In addition to the deracemization of amines, biocataytic applications of *A. niger* MAO have also included the desymmetrization of substituted pyrrolidines and the enantioselective oxidation of hydroxylamines [14], [15].

We are interested in exploring the potential of bacterial amine oxidases as alternatives to biocatalysts derived from fungal enzymes and knowledge of enzyme structure plays an important role in this process. While a number of MAOs from bacterial sources have been kinetically and chemically characterized, thus far, none have been described from a structural perspective [16], [17]. We present here a structural analysis of a bacterial cyclohexylamine oxidase (CHAO) in both the holoenzyme form with bound flavin adenine dinucleotide (FAD), as well as a ternary complex with FAD and the oxidation product cyclohexanone. Originally described in *B. oxydans* strain IH-35A, CHAO carries out the initial step in the biodegradation pathway of cyclohexylamine, a high volume chemical used industrially as a corrosion inhibitor and in the rubber and water treatment industries [16], [18]. The cyclohexylamine biodegradation pathway leads ultimately to adipate, and includes the enzyme cyclohexanone monooxygenase (CHMO), a Baeyer-Villiger monooxygenase of known structure [19], [20]. The catalytic performance of CHAO was recently investigated, revealing activity towards a range of amine substrates [21]. We compare the structure of CHAO to its closest known structural relatives, highlighting important similarities and differences. In addition, we use random acceleration molecular dynamics (RAMD) to identify possible routes of exit for products from the active site and propose an egress mechanism based on these results.

## Experimental Procedures

### Gene Cloning

To facilitate cloning of the CHAO-encoding (*chaA*) gene, internal peptide sequences of the purified CHAO from *B. oxydans* strain IH-35A were determined using an automated protein sequencer (Perkin Elmer Model 477) following digestion of CHAO with lysyl endopeptidase and separation of fragments by SDS PAGE. Two peptide sequences: ESVTPDPDVD and ALDELADSVG were obtained, with the first sequence being in agreement with the N-terminal amino acid sequence of this enzyme reported previously [16]. Two degenerate primers derived from the amino acid sequence were synthesized and used in PCR amplification of a partial CHAO-encoding gene from the total genomic DNA of strain IH-35A. Before this amplified DNA was used as a hybridization probe, its nucleotide sequence was determined to confirm its authenticity. The amplified fragment was then used to probe a Southern hybridization of digested *B. oxydans* IH-35A genomic DNA. Two fragments that probed positive were cloned and sequenced and found to be overlapping. The DNA fragment-carrying *chaA* was amplified using *Pfu* DNA polymerase (Stratagene), digested with *Eco* RI and *Pst* I and cloned into linearized pSD80 vector. The result was the CHAO expression vector pCA101. The DNA sequence of CHAO, together with adjacent sequences (5.3-kb) has been submitted to DDBJ/EMBL/GenBank (accession number: AB698640).

### Expression and Purification of Recombinant CHAO

*E. coli* BL21 cells harboring the CHAO expression vector pCA101 were grown in 100 mL of LB medium containing 100 µg/mL of ampicillin at 30°C. At an A_600_ of 0.5, isopropyl β-D-1-thiogalactopyranoside (IPTG) was added to a final concentration of 0.5 mM and the cells cultured for an additional three hours. Following harvesting by centrifugation, the cells were washed with buffer (50 mM phosphate, pH 6.5), resuspended in buffer and sonicated in three 30-second bursts. Following sonication, the lysate was cleared by centrifugation for 1 h at 48,000 g and 4°C. CHAO activity was assayed by measuring the rate of oxygen consumption as described previously [16]. One unit of CHAO was defined as the amount that consumed 1 µmol of oxygen per min under assay conditions. Protein concentration was determined by method of Bradford using crystalline bovine serum albumin as the standard [22].

All enzyme purification steps were performed at 4°C. The crude extract obtained from approximately 2.5 g of cells (wet weight) was fractionated with ammonium sulfate, and the fraction at 20–40% saturation was retained. After dialysis of the retained fraction against buffer, the protein solution was loaded on a DEAE Cellulose DE-52 column (2.5×6.9 cm) equilibrated with buffer. After washing the column with additional buffer, the enzyme was eluted from the column using a 252 mL linear gradient of buffer with the addition of 0–0.5 M NaCl (flow rate of 21 mL/h). The CHAO protein eluted at 0.34–0.38 M NaCl. The active fractions (21 mL total) were pooled and dialyzed against buffer. Ammonium sulfate was added to the enzyme fraction to a final concentration of 10% before it was put on a Toyopearl Butyl-650S (Tosoh Bioscience) column equilibrated with buffer with the addition of 10% ammonium sulfate. The flow through fraction was collected and dialyzed against buffer.

Additional information relating to the cloning, expression and purification of the CHAO enzyme has been provided as (Text S1, Figure S1, Table S1 and Text S2).

### Protein Crystallization and Structure Solution

Crystallization of the CHAO holoenzyme complex was carried out using the hanging drop vapor diffusion method. Crystals were grown with 30% PEG 8000 and 0.2 M sodium acetate trihydrate in 0.1 M sodium cacodylate pH 6.5. Drops contained 2 µL of purified protein (10 mg/mL) in 50 mM Tris pH 8.5 mixed with 2 µL of well solution. Crystals of the ternary complex were obtained by incubating protein overnight in five molar excess cyclohexanone prior to setting up crystallization experiments. The crystallization precipitant used was 1.6 M ammonium sulfate, 10% v/v dioxane and 0.1 M MES pH 6.5. Mineral oil and Paratone were used as cryoprotectants for both crystal forms. Diffraction data were collected on beamline X8C of the National Synchrotron Light Source (Upton, NY) under cryogenic conditions (−180°C) using an ADSC Quantum 4R CCD detector. Data processing for all data sets was carried out using the HKL software suite [23]. Phasing of the holoenzyme structure was accomplished by molecular replacement as implemented in the software package PHASER, with the monomeric structure of human MAO B (PDB: 1GOS) serving as the search model [5], [24]. For the ternary complex, the refined holoenzyme structure was used as the search model for molecular replacement using PHASER. Both structures were refined using strict four-fold non-crystallographic symmetry (NCS) and included simulated annealing, energy minimization and restrained B-factor refinement as implemented in the CNS program suite [25]. Manual model building was carried out with the program PyMOL [26]. Towards the end of refinement, NCS constraints were relaxed to restraints but monitoring of the R_free_ suggested that the data to parameter ratio had been exceeded and thus, the individual molecules in the asymmetric unit were not refined separately. Given the limited resolution of the data no solvent molecules were added.

### Molecular Dynamics Simulations

As presented below, no obvious ingress/egress route for substrate/product could be identified in the CHAO structure. In order to identify possible routes for cyclohexanone to exit the CHAO active site, the ternary complex with FAD and cyclohexanone was analyzed using the random accelerated molecular dynamics method (RAMD) [27]. The simulation of ligand egress from buried active sites is a significant computational undertaking, since the time scale over which the process takes place can be substantially greater than that which can be simulated using classical molecular dynamics methods. The RAMD method addresses this problem by applying a small, randomly oriented force to the ligand, increasing the probability of ligand egress in a computationally accessible simulation time [27]. The ligand is thus able to unbiasedly search for exit routes over a relatively short time scale, without the requirement to specify an initial search pathway or direction. Parameters were assigned for amino acid residues using the CHARMM27 force field [28], [29]. CHARMM force field parameters for the FAD and cyclohexanone molecules were generated using the CgenFF web server (http://mackerell.umaryland.edu/~kenno/cgenff/). The complex was solvated in a water sphere, resulting in a total of 20779 atoms in the simulation system.

All molecular dynamics simulations were carried out using the NAMD software package [30]. Before beginning the RAMD simulation, the system was first minimized and equilibrated for approximately 500 ps. Once the system was equilibrated, 35 RAMD simulation runs were carried out to identify potential egress routes for cyclohexanone. Each run consisted of a maximum 2 ns simulation, with the acceleration force (accel) set to 0.10 Kcal/mol*Å*amu (default 0.25 Kcal/mol*Å*amu) and the distance threshold parameter (rMinRamd) set to 0.005 Å (default 0.01 Å). All remaining RAMD parameters were assigned default values. The Langevin dynamics method was used for temperature control, and the spherical harmonic boundary condition method was used to contain atoms in the water sphere during the simulations.

## Results

### Expression of chaA Gene in *E. coli*

Overexpression of CHAO in an IPTG-inducible plasmid pCA101 derived from pSD80 was estimated to be 20% of the total cellular protein. The specific activity of CHAO in crude extract of *E. coli* [pCA101] with cyclohexylamine as substrate was found to be 5.9 U/mg, an 11-fold improvement over the *Brevibacterium* wild-type protein activity [16]. The enzyme from the cell extract was purified 5-fold with approximately 20% yield after three steps. The purified enzyme showed a single protein band on both native PAGE and SDS-PAGE (not shown). Gel filtration on Superose 12B showed a *M*_r_ of 50 kDa, indicating CHAO to be a monomeric protein like the parental enzyme from *Brevibacterium*.

The DNA-deduced amino acid sequence of *B. oxydans* CHAO consists of 488 residues, with a predicted signal peptide 23 residues in length. Consequently, the N-terminus of the *B. oxydans* CHAO (ESVTPDPDVD) corresponds to residues 24–33 of the DNA-deduced sequence. The N-terminal sequence of the recombinant CHAO expressed in *E. coli* was determined to be THLNTYESVTPDPDVDVI, corresponding to residues 18–35 of the DNA-deduced sequence. The recombinant enzyme is 6 residues longer at the N-terminus, possibly due to differences in the signal peptide cleavage sites in *E. coli* and *B. oxydans*.

### Crystallization and Structure Solution

Generally, crystal formation would occur after 1–2 days of diffusion equilibration. Crystals were of a bright yellow color when viewed under a non-polarizing microscope, suggesting the presence of the FAD cofactor. Difficulties were encountered during data collection, as no effective cryoprotectant for the CHAO crystals could be identified. An exhaustive survey of saccharides, PEGs and other additives was met with limited success in that the formation of ice rings prevented the collection of high-resolution data. Successful data collection under cryogenic conditions was ultimately achieved using mineral oil and Paratone. However, the data sets for both the holoenzyme and ternary form were still subject to high anisotropy and large mosaic spread (approx. 3°). In order to determine the holoenzyme structure of CHAO, we employed molecular replacement using the A chain from human MAO B (30% sequence identity), truncated to remove the C-terminal predicted transmembrane helical segment, as a search model. The structure was solved using improved maximum likelihood based molecular replacement algorithms in the program PHASER [24]. Four molecules of CHAO were identified per asymmetric unit. The structure of the CHAO·FAD·cyclohexanone ternary complex was solved by molecular replacement using the structure of the holoenzyme monomer as a search model. As with the holoenzyme, the ternary complex crystallized with four molecules per asymmetric unit. Statistics associated with data processing and structure refinement are summarized in Table 1.

**Table 1:** Data Collection and Refinement Statistics for CHAO Holoenzyme and Ternary Complexes.

	CHAO·FAD	CHAO·FAD·Cyclohexanone
**Data Collection**		
Space Group	C222_1_	P2_1_2_1_2_1_
Cell Dimensions (Å)	a = 217.7, b = 234.6, c = 90.1	a = 86.5, b = 138.5, c = 177.2
Resolution^a^(Å)	50.00–3.00 (3.11–3.00)	50.00–2.93 (3.03–2.93)
Completeness (%)	95.8 (92.8)	95.1 (95.3)
R_merge_^b^ (%)	14.3 (40.3)	11.8 (28.8)
I/σ(I)	16.0 (5.5)	14.9 (4.3)
Wilson B-factor (Å^2^)	32.1	26.0
Number of Reflections	38, 366	42,275
**Refinement**		
R_cryst_^c^	24.0	23.1
R_free_^d^	25.3	26.2
Molecules per A.U.	4	4
Number of Atoms^e^	3,517	3,524
RMSD bonds (Å)	0.01	0.01
RMSD angles (°)	1.54	1.80
B-factors (Å^2^)		
Overall	26.4	22.5
Protein	26.4	22.5
Cofactor	26.5	18.3
Product	N/A	49.0

a Numbers in brackets represent data from the highest resolution shell.

b
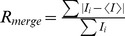
, where 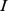 represents the intensities of a multiply measured reflection hkl and 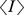 their average.

c
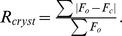

d R_free_ is the R-factor calculated from 10% of the data not included in refinement.

e The number of atoms refined is reduced by a factor of four as strict four-fold non-crystallographic symmetry was employed during refinement.

### Overall Structure

The crystal structures of CHAO in the holoenzyme form and as a ternary complex with FAD and cyclohexanone were determined to a resolution of 3.00 Å and 2.93 Å, respectively. The enzyme was observed to be monomeric, consistent with our size exclusion chromatography results. The first amino acid residue observed in the CHAO structure corresponds to residue 30 of the DNA-deduced *B. oxydans* sequence. The first twelve residues of the recombinant CHAO sequence (residues 18–29 of the DNA-deduced sequence) are not observed in either the holoenzyme or ternary complex electron density maps. This is likely due to disorder in these residues as a result of their position at the flexible terminus of the polypeptide chain. Most of the polypeptide chain could be modeled into the electron density maps: residues 30–480 in the case of the holoenzyme and residues 30–478 in the ternary complex. In both structures, well-defined electron density was observed for one FAD cofactor per protein molecule. Several amino acid residues were modeled as alanine residues when no significant electron density was observed for their side chains. For the apoenzyme structure, 90% of the residues fall into the favored region of a Ramachandran plot and 2.2% are outliers. In the structure of the ternary complex, 94% are in the favored region and 0.7% are outliers. Residues that lie outside the favored region were located in flexible surface loops.

**Figure 1:**
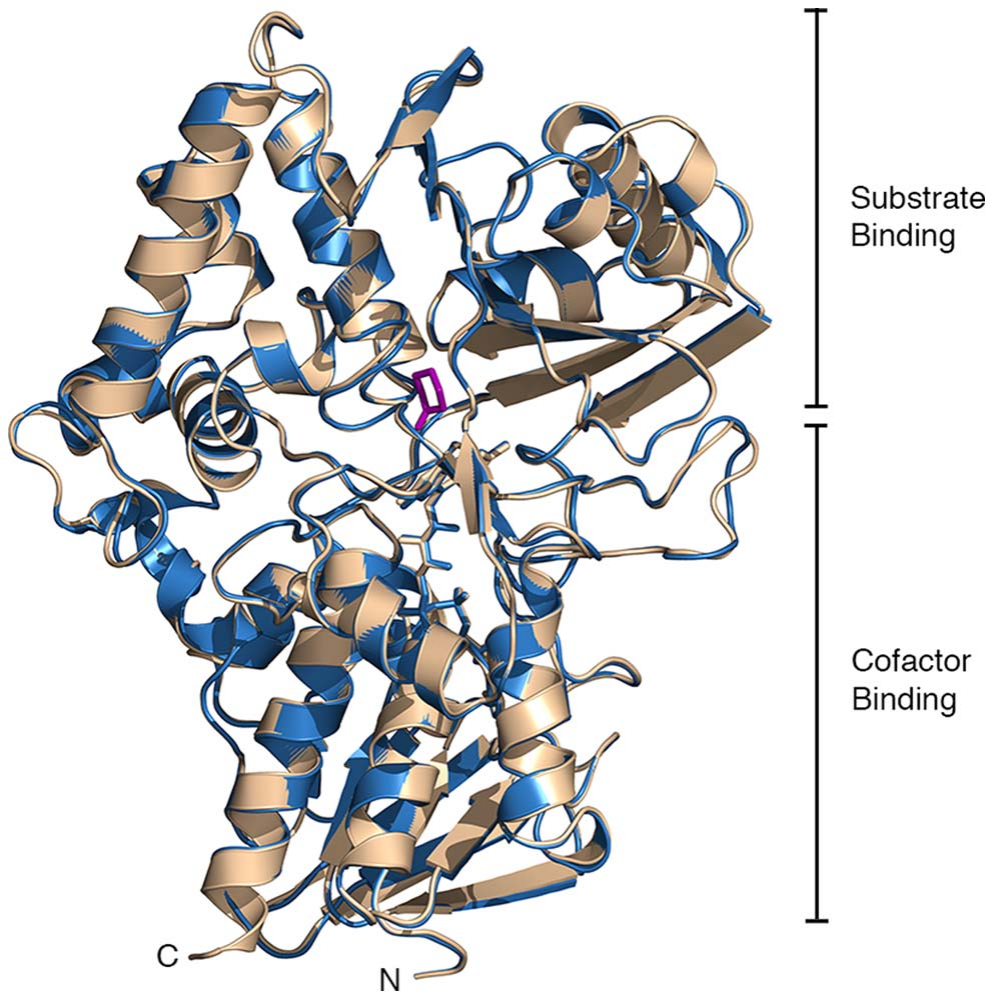
The tertiary structure of CHAO. A cartoon representation of the superimposed holoenzyme (wheat) and ternary complex (blue) forms of CHAO. The FAD cofactor and cyclohexanone molecule (magenta) are shown as sticks. The cofactor- and substrate-binding domains are labeled.

The CHAO structure can be divided into two domains: a cofactor-binding domain composed of two four-stranded β-sheets (one parallel, one mixed) and seven α-helices, and a substrate-binding domain consisting of a twisted seven-stranded mixed β-sheet and eight α-helices. The general topology of CHAO is known as the PHBH fold, after *p-*hydroxybenzoate hydroxylase – a flavoenzyme from *Pseudomonas fluorescens* in which the topology was first described [31]. This fold has since been observed in a number of other flavoenzymes, including cholesterol oxidase, polyamine oxidase and monoamine oxidase [5], [32], [33]. The core structure of the cofactor-binding domain is a four-stranded parallel β-sheet, sandwiched between a four-stranded mixed β-sheet on one side and four α-helices on the other. The ADP-ribityl moiety of FAD is embedded within the domain, with the isoalloxazine ring of the cofactor extending towards the interface of the cofactor-binding and substrate-binding domains. A notable feature of the substrate-binding domain is a βαβ unit of supersecondary structure (residues 34–60) containing the GXGXXG phosphate-binding consensus sequence. The substrate-binding domain is dominated by the seven-stranded mixed β-sheet. This β-sheet faces the isoalloxazine ring of FAD where it forms part of the active site wall. When superimposed, the structures of CHAO crystallized with and without substrate present are very similar (RMSD 0.26 Å), with no significant differences in the positioning of secondary structural elements and with only minor differences in the conformation of loop regions (Figure 1).

### Substrate Binding Site

**Figure 2:**
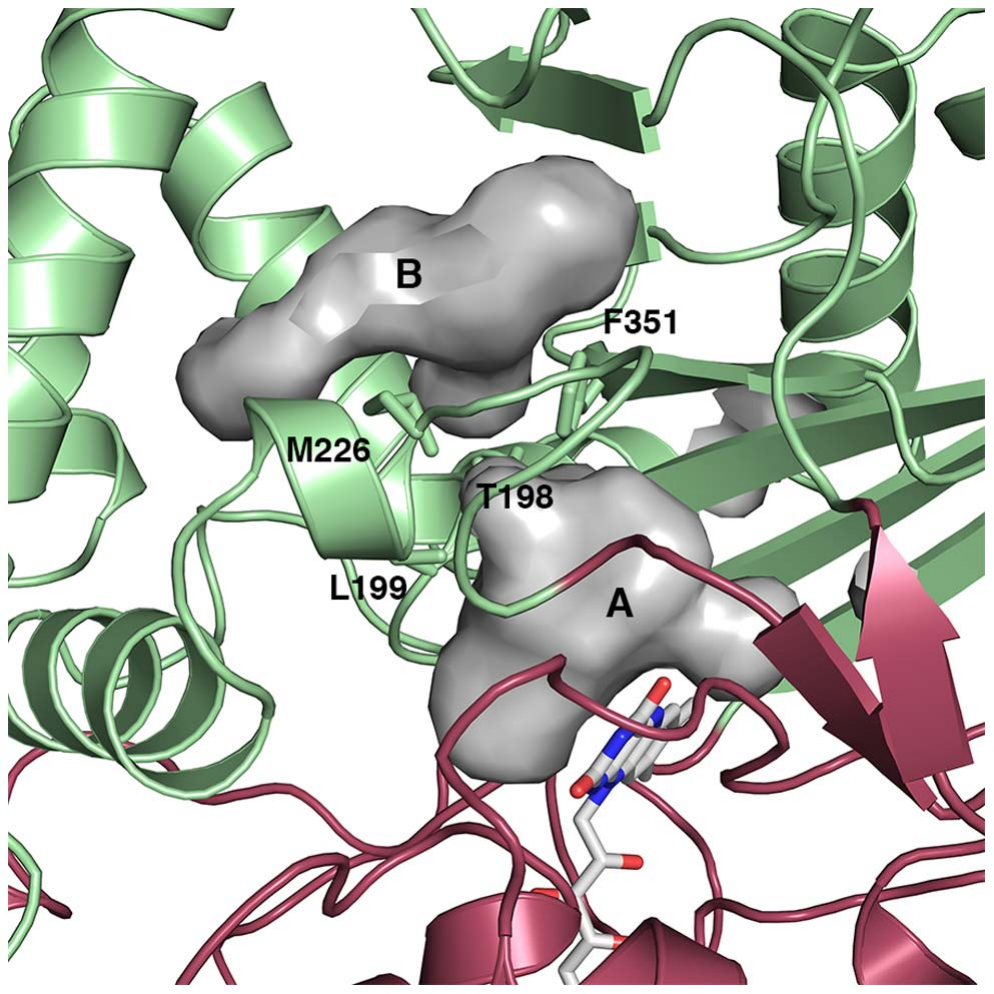
Access to the CHAO active site. An illustration of the substrate-binding (A) and secondary (B) cavities in the CHAO structure. The protein is shown as a cartoon figure, with the substrate-binding domain colored green and the cofactor-binding domain colored red. FAD is shown as sticks, as are four residues that separate the two cavities (T198, L199, M226, and F351). Cavities were visualized with the program PyMOL using the default 1.4 Å probe radius.

The active site of CHAO is located in a buried cavity located at the interface of the substrate- and cofactor-binding domains (Figure 2). The cavity is largely hydrophobic in character as a consequence of the number of aromatic and aliphatic amino acids lining its periphery. These include the prominent aromatic side chains of F88, Y215, Y321, F368 and Y459. One side of this cavity is occupied by the flavin cofactor, held in place by hydrogen bonds and Van der Waals interactions with the protein. The isoalloxazine ring of the FAD cofactor is planar, and a lysine residue that is hydrogen bonded to the N5 atom of FAD in other MAOs (*via* a water molecule) is positioned to play that role in CHAO. In addition to the substrate-binding cavity, CHAO has a second cavity located closer to the protein surface that is shielded from the bulk solvent by a loop of the polypeptide chain (residues 128–138). This intermediate cavity is separated from the substrate-binding cavity by a series of amino acid side chains (T198, L199, M226, and F351).

**Figure 3:**
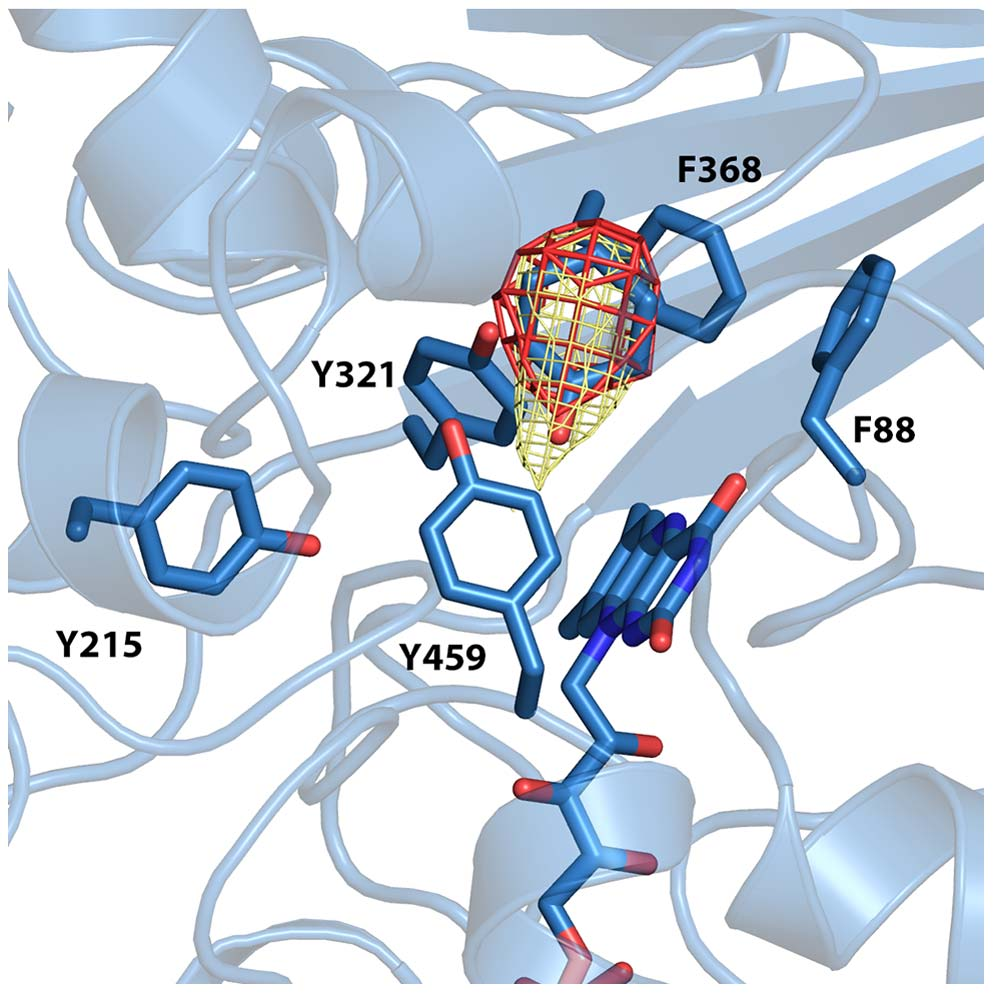
The CHAO substrate-binding pocket. A depiction of the CHAO substrate-binding pocket, highlighting conserved aromatic side chains. The CHAO ternary complex is shown as a blue cartoon, with the conserved side chains, cofactor and cyclohexanone molecule shown as sticks. The Fo-Fc electron density before the cyclohexanone molecule was included in refinement is shown as red mesh (contoured at 1.5 σ). The final 2Fo-Fc electron density for the cyclohexanone is shown as yellow mesh (contoured at 1σ).

An examination of an omit map in the area of the substrate binding pocket for CHAO crystallized in the presence of excess cyclohexanone shows an appropriately-shaped positive electron density feature large enough to accommodate a cyclohexanone molecule (Figure 3). The peak is relatively weak however, indicating that the occupancy of the active site by cyclohexanone is not 100%. This is supported by the higher B-factors observed for the ligand atoms as compared to those of the protein (Table 1). Modeling of cyclohexanone into this electron density results in the plane of the cyclohexanone ring being oriented perpendicular to the flavin ring, with the oxygen atom rotated towards the FAD cofactor at a distance of 3.7 Å from the flavin N5 atom. In this position, the cyclohexanone molecule is sandwiched between the aromatic side chains of Y321, F368 and Y459. These residues, along with the cofactor, form an “aromatic cage” similar to that observed in other MAO structures.

### Random Acceleration Molecular Dynamics Simulations

**Figure 4:**
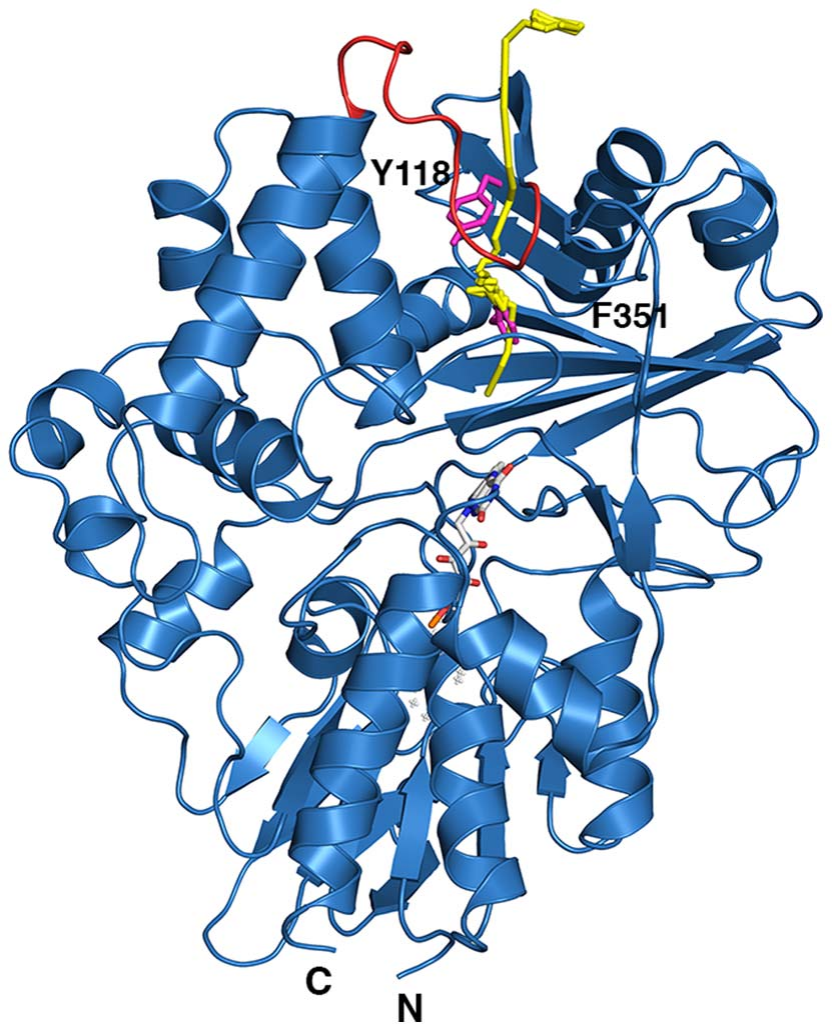
The proposed CHAO substrate egress route. Cartoon figure of CHAO (blue) showing the principal egress route for cyclohexanone identified by RAMD (yellow). The flexible loop covering the entrance cavity (residues 118–128) is colored red and two important residues along the egress path (F351 and Y118) are shown as magenta sticks. The FAD cofactor is shown as sticks.

The preliminary energy minimization and molecular dynamics equilibration steps resulted in a protein that was of stable structure (Figure S2). The equilibrated structure was similar to the crystal structure of the ternary complex and regions of the protein identified as most flexible in the molecular dynamics simulations corresponded to parts of the crystal structure with highest B-factors (Figure S3). A total of 35 RAMD simulations were carried out, representing a total simulation time of 22.8 ns. Of the 35 simulation runs, four were not able to identify an egress route for cyclohexanone from the CHAO active site within the 2 ns simulation time. In the simulations that were able to identify egress routes, the time required for cyclohexanone to leave the CHAO active site and reach the protein surface varied from 20 ps to approximately 2 ns. The results indicate that one path out of the protein is favored (Figure 4). This route involves the cyclohexanone diffusing from the substrate-binding pocket to the intermediate cavity and from there to the protein surface. There are two steps involved in cyclohexanone egress, each involving a localized change in conformation in the protein in order to allow the molecule to proceed. First, the side chain of F351 must rotate and permit cyclohexanone to travel from the substrate-binding pocket to the intermediate cavity. The second required event is a change in conformation of the side chain of Y118 and a concomitant movement of the flexible loop covering the intermediate cavity. This allows the product to leave the intermediate cavity and be released into the bulk solvent.

## Discussion

### Overall Structure

An examination of the three dimensional atomic structure of CHAO reveals that the overall fold is substantially similar to that of the human MAO B monomer, with a RMSD of 1.1 Å for the coordinates of the α-carbon atoms. Given the level of sequence similarity with human MAO B (30% identity), the structural similarity between it and CHAO is not surprising. Like MAO B, the CHAO structure is composed of two domains - a substrate-binding region and a cofactor-binding domain, with the isoalloxazine moiety of FAD at their interface. RMSD values for α-carbon atoms range from a low of 0.1 Å adjacent to the FAD cofactor, to a maximum of 7.9 Å at one end of the largest helix in the substrate-binding domain. The most obvious structural difference between CHAO and MAO B is found at the C-terminus, where MAO B possesses a significant extension (∼50 residues) not found in CHAO. This extension forms a predicted transmembrane helical segment important in anchoring MAO B to the outer mitochondrial membrane [5]. The polypeptide backbone in the remainder of the protein is remarkably similar in the two proteins, with minor differences in the orientations of secondary structural elements and the conformations of the loops between these elements. Other related enzymes, such as human MAO A, also have significant C-terminal membrane anchoring extensions that are absent in the CHAO structure. CHAO is not the only member of this family of enzymes without a C-terminal tail however. MAO N from *A. niger* has a C-terminus much like that of CHAO, consistent with its proposed localization to the peroxisome and not a membrane [34]. While similar to CHAO in its lack of a C-terminal membrane anchor, the structure of MAO N has a significant difference in the form of a large (40 residues) N-terminal extension of unknown function. Possessing neither C- nor N-terminal extensions, the CHAO structure is the smallest known member of this group of MAOs.

### Substrate Binding Site

The walls of the CHAO substrate-binding pocket are lined with aromatic and aliphatic residues. Prominent aromatic side chains such as F88, Y215, Y321, F368 and Y459 contribute to a hydrophobic environment favorable for the binding of apolar monoamine substrates. Many of these residues are highly conserved in the structures of CHAO and human MAO A, MAO B and MAO N and if not conserved, are replaced by residues of similar size and chemical properties. In the CHAO structure, the side chains of Y321, F368 and Y459, along with the isoalloxazine moiety of the flavin, form a cage-like structure in which the cyclohexanone is bound. This arrangement is similar to that observed in structures of MAO B in which inhibitors bind between the side chains of Y398 and Y435 [35]. The observation of an “aromatic cage” in MAO B and the structures of a number of other flavin-dependent amine oxidizing enzymes suggests a basic functional role for this feature. Studies indicate that the cage does not play a significant role in maintaining the structure of the active site, but instead serves to orient the amine substrates and promote catalysis [36].

**Figure 5:**
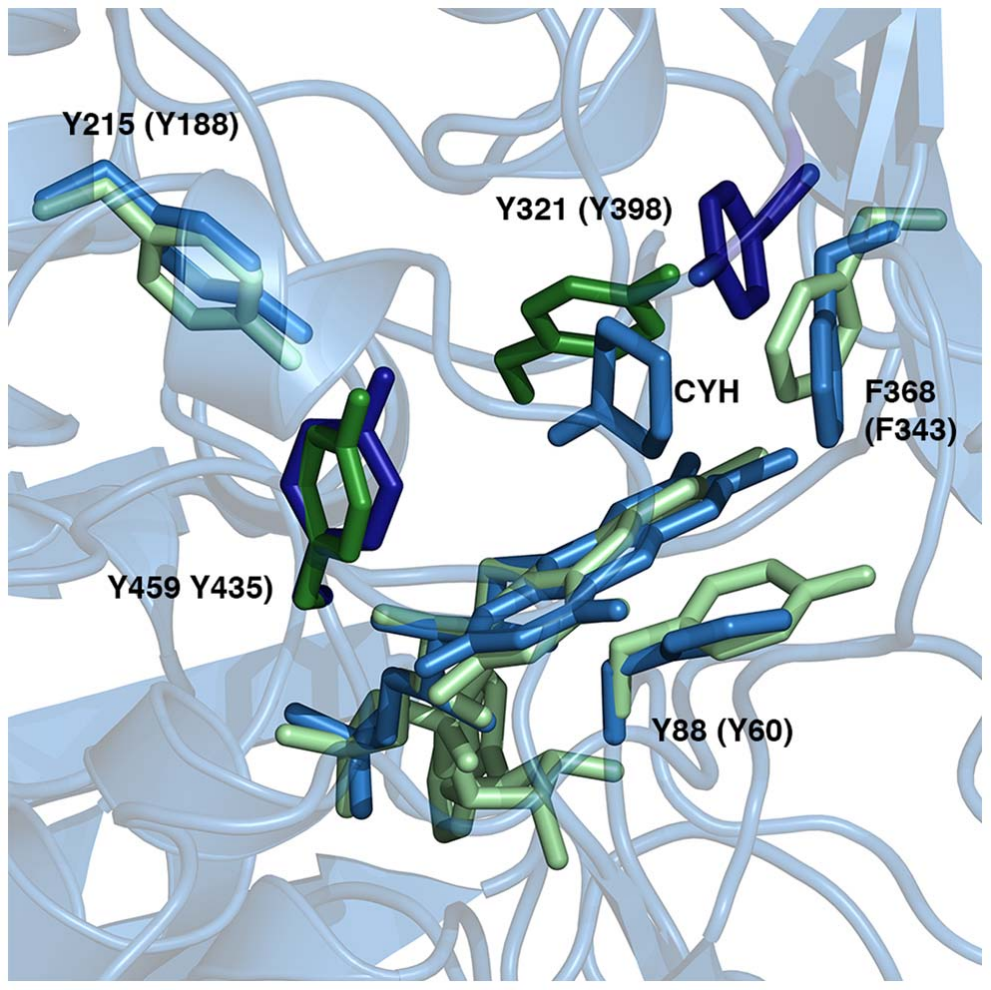
Conserved aromatic residues in the active sites of CHAO and MAO B. The CHAO structure is shown as a blue cartoon with conserved residues shown as sticks, with the conserved residues in MAO B shown as green sticks. The two side chains that form the aromatic cage with the FAD cofactor are highlighted with darker shades of color. The residue number of the side chains in the CHAO structure is shown, with the corresponding residue number in the MAO B structure in parentheses. CYH is cyclohexanone.

While the components of the aromatic cage are conserved in MAO B and CHAO, there are differences. The orientation of one of the conserved residues (Y321 in CHAO) is significantly different than its counterpart in the MAO B structure (Figure 5). While the side chains of the two residues are oriented such that their hydroxyl groups are located in the same position in space, the residues themselves are located in divergent locations in the protein sequence and the aromatic rings of the side chains do not overlap. The conformation of Y321 in CHAO results in a more open aromatic cage than that observed in MAO B. In contrast to the structure of the aromatic cage of MAO B, the side chains of Y368 and Y459 in CHAO are approximately 10 Å apart and are not coplanar with the cyclohexanone ring. It is not clear that these residues could perform a similar role as proposed for MAO B. In the case of CHAO, a third aromatic residue in the substrate-binding site may also be important. The side chain of F368 is positioned near the cyclohexanone molecule, with its aromatic ring coplanar with the six-membered ring of cyclohexanone. In this position, it may be in a more suitable position to play a functional role in the mechanism of CHAO than Y321. This unusual orientation of one of the residues of the aromatic cage appears to be unique to CHAO. The geometry of the aromatic cage in MAO A is very similar to that of MAO B, differing from CHAO in the orientation the tyrosine residue. Interestingly, the aromatic cage of MAO N differs from CHAO, MAO A and MAO B in that one of the residues involved is a tryptophan instead of tyrosine. While the identity of the residue is different in MAO N, the geometry of the aromatic cage of MAO N is similar to that of MAO A and MAO B. Only in CHAO do we observe the atypical orientation resulting in the more open aromatic cage.

### Substrate Entry/Exit

Since the active site of CHAO is located in the interior of the protein and inaccessible to bulk solvent, possible routes of substrate entrance and product exit are not obvious. Substrate entrance and product exit would seem to require either a clamshell-like movement of the substrate- and cofactor-binding domains, or a more localized movement of flexible secondary structural elements. The structures of the CHAO holoenzyme and complex with cyclohexanone are remarkably similar, suggesting that there are no significant conformational changes associated with the binding of product in the active site. A random acceleration molecular dynamics simulation was carried out in order to identify flexible regions of the CHAO structure and to find potential routes of product egress. This enhanced sampling technique has been used to study substrate and product release pathways in other systems, including cytochrome P450 and rhodopsin [27], [37].

The RAMD simulations performed using the structure of CHAO with cyclohexanone bound in the substrate-binding pocket identified one route of egress as predominant. This route involved a two-step process in which the cyclohexanone molecule diffuses out of the substrate-binding pocket into a second smaller cavity, followed by a second step in which the molecule leaves this intermediate cavity and enters the bulk solvent. The bipartite nature of the substrate-binding cavity is not unique among the family of MAOs to which CHAO belongs. A similar two-part cavity is also observed in human MAO B for which a mechanism for substrate entry has been proposed that shares a number of features with the mechanism we have proposed for CHAO. As with CHAO, substrate entry is believed to be modulated by a shielding loop of protein structure, and subsequent entry into the active site depends on the transient movement of residues separating the entrance and active site cavities [35].

Despite the similarities between the two part cavities observed in CHAO and MAO B, this feature is not observed in the structures of the other members of this family of enzymes. In contrast to the two cavities observed in the structures of CHAO and human MAO B, the structure of human MAO A contains a single substrate-binding cavity separated from the protein surface by a loop of protein structure. In the structure of the fungal MAO N, the shielding loop is completely absent and is instead replaced by an α-helix. As a consequence, a large cavity, lined with hydrophobic residues, extends from the surface of MAO N to the substrate-binding site. Thus, in contrast to the significant similarity observed between these enzymes in the arrangement of important active site residues, there is considerable diversity in the route to and from the active site.

MAOs have been the subject of considerable interest due to their medically important role in controlling amine neurotransmitter levels and because of their potential for use as applied biocatalysts. Our knowledge of the structure of this group of enzymes has been limited to a small number of examples including the structures of human MAO A and B, and to MAO N from *A. niger*. The structure of CHAO from *B. oxydans* IH-35A presented here provides additional structural insight into this group of enzymes, revealing the degree of conservation of fundamental active-site features as well as the diversity of the substrate-binding cavities. It provides a first look at the structure of a small, soluble MAO that provides a useful structural framework for further studies, including mutagenesis directed towards understanding mechanism and substrate specificity as well as the engineering of this amine oxidases for use as a practical biocatalyst.

### Accession Numbers

The DNA sequence of CHAO, together with adjacent sequences (5.3-kb) has been submitted to DDBJ/EMBL/GenBank (GenBank accession number **AB698640**). Atomic coordinates have been deposited with the Worldwide Protein Data Bank (PDB ID: **4I58** and **4I59**).

## Supporting Information

Figure S1 **CHAO gene context.**
(DOCX)

Figure S2 **RMSD of C_α_ coordinates during molecular dynamics equilibration.**
(DOCX)

Figure S3 **Comparison of equilibrated molecular dynamics and crystal structures.**
(DOCX)

Table S1 **Summary of ORF characteristics.**
(DOCX)

Text S1 **Additional information relating to cloning and expression.**
(DOCX)

Text S2 **Sequence characteristics.**
(DOCX)
