# Supplementary material for: Structural Analysis of a Novel Cyclohexylamine Oxidase from Brevibacterium oxydans IH-35A
Source: PLoS One. 2013 Mar 26;8(3):e60072. doi: 10.1371/journal.pone.0060072 (PMC3608611; doi:10.1371/journal.pone.0060072)
Supplement: Figure S1 — CHAO gene context. (DOCX) [file pone.0060072.s001.docx]

**Figure S1.** **CHAO gene context.** Cloning and sequence analysis of the 5.3-kb DNA locus of strain IH-35A produced 4 open reading frames. The 1464-bp ORF preceded by a consensus ribosome binding sequence (AGG) in pCA200 was identified as encoding CHAO. CHAO is likely regulated by a 190 amino acid protein (ChaR) belonging to the AsnC/Lrp–type family of transcriptional activators and transcribed in an opposite DNA strand upstream of *chaA* separated by a 1044-bp intergenic space. Downstream of *chaR*, in the same direction and separated by a 355–bp intergenic sequence is an incomplete ORF designated *chaP1*; and also downstream of *chaA* in the same direction and separated by a 547-bp intergenic sequence is a partial ORF, designated *chaP2*.
