# Supplementary material for: Structural Analysis of a Novel Cyclohexylamine Oxidase from Brevibacterium oxydans IH-35A
Source: PLoS One. 2013 Mar 26;8(3):e60072. doi: 10.1371/journal.pone.0060072 (PMC3608611; doi:10.1371/journal.pone.0060072)
Supplement: Figure S2 — RMSD of Cα coordinates during molecular dynamics equilibration. (DOCX) [file pone.0060072.s002.docx]

**Figure S2. RMSD of C_α_ coordinates during molecular dynamics equilibration.** Before the RAMD simulations were carried out, the system was subjected to energy minimization (5000 steps) to insure that there were no steric clashes or inappropriate geometry. Minimization was followed by an approximately 500 ps molecular dynamics equilibration step. The plot of RMSD (C_α_ atoms) versus time for this simulation plateaus after 300 ps, indicating that the structure has equilibrated.
