# Supplementary material for: Structural Analysis of a Novel Cyclohexylamine Oxidase from Brevibacterium oxydans IH-35A
Source: PLoS One. 2013 Mar 26;8(3):e60072. doi: 10.1371/journal.pone.0060072 (PMC3608611; doi:10.1371/journal.pone.0060072)
Supplement: Figure S3 — Comparison of equilibrated molecular dynamics and crystal structures. (DOCX) [file pone.0060072.s003.docx]

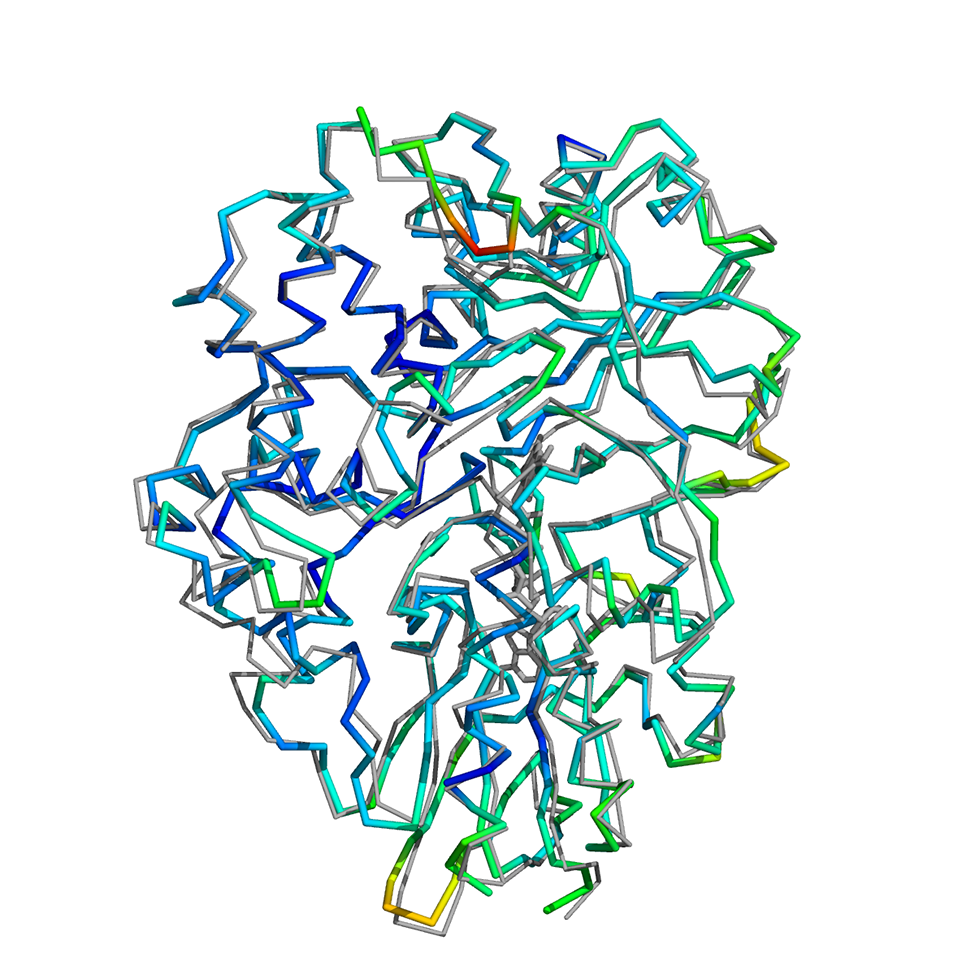


**Figure S3. Comparison of equilibrated molecular dynamics and crystal structures.** A superposition of the CHAO coordinates following the MD equilibration step and those of the crystal structure is shown. The backbone of the equilibrated molecular dynamics structure is shown as a grey ribbon, while that of the ternary complex crystal structure is shown in color. The crystal structure is colored according to crystallographic B-factor, with the blue end of the spectrum indicating the regions of lowest B-factors and red indicating those with the highest values. The two structures are highly similar (RMSD 1.0 Å for backbone atoms), with the largest differences found in surface loops between secondary structural elements. These loops also possess, by and large, higher B-factors in the crystal structure. One end of an α-helix at the protein surface shows a slight difference between the equilibrated model and the crystal structure, perhaps due to the influence of crystal packing or the adjacent flexible loop. The region of the crystal structure with the highest B-factors is the surface loop shielding the proposed access route to and from the active site. This is consistent with the results of the molecular dynamics simulations, which suggest that this area of the structure is highly flexible.
