# Supplementary material for: Structural Analysis of a Novel Cyclohexylamine Oxidase from Brevibacterium oxydans IH-35A
Source: PLoS One. 2013 Mar 26;8(3):e60072. doi: 10.1371/journal.pone.0060072 (PMC3608611; doi:10.1371/journal.pone.0060072)
Supplement: Table S1 — Summary of ORF characteristics. (DOCX) [file pone.0060072.s004.docx]

**Table S1.** **Summary of ORF Characteristics.**

| Protein | Amino acids | Homology search (BLAST Feb. 2012) |
| --- | --- | --- |
| ChaA | 488 | monoamine oxidase A [*Crassostrea gigas*] (Pacific oyster) CAD89351 521 aa  Identities = 166/464 (35.8%), Positives = 261/464 (56.3%), Gaps = 12/464 (2.6%)  putative monoamine oxidase [*Azospirillum* sp. B510] (bacteria) BAI75019 458 aa  Identities = 161/446 (36.1%), Positives = 244/446 (54.7%), Gaps = 4/446 (0.9%)  putative monoamine oxidase [*Branchiostoma floridae*] (Florida lancelet) EEN49056 512 aa  Identities = 164/458 (35.8%), Positives = 248/458 (54.1%), Gaps = 12/458 (2.6%)  putative monoamine oxidase [*Gordonia araii* NBRC 100433] (bacteria) GAB10041 452 aa  Identities = 166/447 (37.1%), Positives = 242/447 (54.1%), Gaps = 1/447 (0.2%) |
| ChaR | 190 | putative transcriptional regulator, AsnC family [*Tsukamurella paurometabola* DSM 20162] ADG80523 174 aa  Identities = 77/161 (47.8%), Positives = 114/161 (70.8%), Gaps = 1/161 (0.6%)  putative transcriptional regulator, AsnC family [*Rhodococcus erythropolis* SK121] EEN84770 173 aa  Identities = 58/141 (41.1%), Positives = 89/141 (63.1%), Gaps = 0/141 (0%)  putative transcriptional regulator, AsnC family [*Rhodococcus erythropolis* PR4] BAH31584 173 aa  Identities = 58/141 (41.1%), Positives = 89/141 (63.1%), Gaps = 0/141 (0%) |
| ChaP1 | >175 | putative amino acid permease [*Pseudonocardia dioxanivorans* CB1190] AEA25918 487 aa  Identities = 73/157 (46.5%), Positives = 93/157 (59.2%), Gaps = 5/157 (3.2%)  putative amino acid permease [*Rhodococcus jostii* RHA1] ABG94414 496 aa  Identities = 73/160 (45.6%), Positives = 95/160 (59.4%), Gaps = 4/160 (2.5%)  putative amino acid permease [*Streptomyces venezuelae* ATCC 10712] CCA60067 482 aa  Identities = 64/152 (42.1%), Positives = 94/152 (61.8%), Gaps = 1/152 (0.7%) |
| ChaP2 | >266 | putative amino acid permease [*Rhodococcus opacus* B4] BAH47146 500 aa  Identities = 114/257 (44.4%), Positives = 165/257 (64.2%), Gaps = 7/257 (2.7%)  putative amino acid permease [*Pseudonocardia dioxanivorans* CB1190] AEA25918 487 aa  Identities = 120/250 (48.0%), Positives = 160/250 (64.0%), Gaps = 8/250 (3.2%)  putative amino acid permease [*Arthrobacter aurescens* TC1] ABM10643 498 aa  Identities = 113/238 (47.5%), Positives = 156/238 (65.5%), Gaps = 2/238 (0.8%) |
