# Supplementary material for: Structural Analysis of a Novel Cyclohexylamine Oxidase from Brevibacterium oxydans IH-35A
Source: PLoS One. 2013 Mar 26;8(3):e60072. doi: 10.1371/journal.pone.0060072 (PMC3608611; doi:10.1371/journal.pone.0060072)
Supplement: Text S1 — Additional information relating to cloning and expression. (DOCX) [file pone.0060072.s005.docx]

**S1.** **Additional information relating to cloning and expression**

Internal peptide sequences of the purified CHAO from *B. oxydans* strain IH-35A were first generated by digestion with lysyl endopeptidase from *Achromobacter lyticus* (Wako Pure Chemical Industries Ltd., Osaka). These peptides were separated by SDS-PAGE (10% polyacrylamide gel) and transferred onto a PVDF membrane using a blotting apparatus (Bio-Rad, Trans-blot SD Semi-Dry Transfer Cell). The protein bands stained with Coomassie Brilliant Blue R-250 were excised and sequenced using an automated protein sequencer (Perkin-Elmer model 477).

To clone the CHAO-containing gene, two degenerate oligodeoxynucleotide primers, derived from amino acid sequence 3 to 9 of chao1 and 3 to 10 of chao2 were synthesized: (5'- GTNACICCNGAYCCIGAYGT -3' and 5'- CCNACISWRTCIGCIARYTCRTC -3'; I=inosine; N=T,C,A or G; R=A or G; Y=C or T; W=A or T) and used in PCR amplification of a partial CHAO encoding gene from total genomic DNA from strain IH-35A prepared by the method of Wilson [[1](#_ENREF_1)]. PCR reactions were performed in a Perkin Elmer-Model 2400 Thermal Cycler for 30 cycles under standard PCR conditions.

Before the amplified DNA (*~*400-bp) was used as a hybridization probe its nucleotide sequence was determined to confirm its authenticity. The digoxigenin-11-UTP labeling system (Roche Molecular Biochemicals) was used to probe a Southern hybridization of strain IH-35A genomic DNA digested with various restriction enzymes (*Bam*HI, *Eco*RI, *Hin*dIII, *Kpn*I, *Nhe*I, *Pst*I, *Sal*I, *Sph*I and *Xba*I). Two fragments (3.9-kb *Sac*I fragment and 2.9-kb *Eco*RI fragment) that probed positive were cloned in *E. coli* XL1-blue using pUC19 as a vector [[2](#_ENREF_2)]. These plasmid derivatives were designated pCA100 and pCA200. Plasmid isolation was performed by the method of Birnboim and Doly [[3](#_ENREF_3)]. Standard procedures such as Southern blot experiment, DNA subcloning, and DNA manipulations were performed by method of Sambrook *et al.* [[4](#_ENREF_4)]. Hybridization was performed at 68°C.

DNA sequencing was determined on both strands by using the *Taq* DyeDeoxy terminator cycle sequencing kit (P.E. Applied Biosystems) and ABI Prism 310 Genetic Analyzer (Perkin-Elmer). The sequence was analyzed using GENETYX-Mac (Software Development Co., Ltd. Chiba, Japan) and the program BLAST [[5](#_ENREF_5)]. Sequencing and analysis found the clones to be overlapping and spanning 5,312-bp.

The DNA fragment carrying *chaA* was amplified by *Pfu* DNA polymerase (Stratagene) with the following pair of PCR primers with the desired restriction sites (*Eco*RI and *Pst*I) to facilitate subsequent cloning (underlined sequences): 5'- CGGAATTCATGTGCCGTAGCAGGCAAGC-3' and 5'- AAAACTGCAGCCATGGAAACGGGAAGG-3'. The amplified fragment was purified from agarose gel, digested with *Eco*RI and *Pst*I and cloned into the linearized pSD80 vector.

**References**

1. Wilson K (2001) Preparation of genomic DNA from bacteria. Curr Protoc Mol Biol Chapter 2: Unit 2 4.

2. Yanisch-Perron C, Vieira J, Messing J (1985) Improved M13 phage cloning vectors and host strains: nucleotide sequences of the M13mp18 and pUC19 vectors. Gene 33: 103-119.

3. Birnboim HC, Doly J (1979) A rapid alkaline extraction procedure for screening recombinant plasmid DNA. Nucleic Acids Res 7: 1513-1523.

4. Sambrook J, Russell DW (2001) Molecular Cloning: A Laboratory Manual: Cold Spring Harbor Laboratory Press.

5. Altschul SF, Madden TL, Schaffer AA, Zhang J, Zhang Z, et al. (1997) Gapped BLAST and PSI-BLAST: a new generation of protein database search programs. Nucleic Acids Res 25: 3389-3402.
