# Supplementary material for: Structural Analysis of a Novel Cyclohexylamine Oxidase from Brevibacterium oxydans IH-35A
Source: PLoS One. 2013 Mar 26;8(3):e60072. doi: 10.1371/journal.pone.0060072 (PMC3608611; doi:10.1371/journal.pone.0060072)
Supplement: Text S2 — Sequence characteristics. (DOCX) [file pone.0060072.s006.docx]

**S2. Sequence characteristics**

The deduced amino acid sequence of CHAO consists of 488 residues with a predicted signal peptide of 23 residues. The predicted size of the mature ChaA (50,334) was in good agreement with the experimental *M*_r_ (50 kDa). Notable sequence features of CHAO are the conservation of a FAD-binding motif (G-G--G) at positions 38-43 and a GG motif (R-GGR--T) at positions 65-72 [[6](#_ENREF_6)]. Like other microbial and plant FAD-containing amine oxidases, the pentapeptide (SGGCY) known to be involved in the covalent binding of FAD to a conserved cysteine residue in human MAO A was absent in CHAO. This suggested that FAD was non-covalently bound in CHAO, supported by the fact that free FAD was extracted easily from denatured purified CHAO protein by ethanol [[7](#_ENREF_7)]. Seven cysteine residues are conserved in the deduced amino acid sequences of animal MAOs, while only C219 is conserved in CHAO.

The homologous proteins of ChaR are involved in amino acid metabolism, transport, or/and biosynthesis. From these observations, we could speculate that ancestor of ChaA was involved in amino acid degradation. The HTH motif of this family is recognized in the N-terminal region, and it corresponds to a position at 35-56 in ChaR sequence [[8](#_ENREF_8),[9](#_ENREF_9)].

Both the partial ORFs of ChaP1 and ChaP2 are likely partial sequences of permeases.

**References**

1. Wilson K (2001) Preparation of genomic DNA from bacteria. Curr Protoc Mol Biol Chapter 2: Unit 2 4.

2. Yanisch-Perron C, Vieira J, Messing J (1985) Improved M13 phage cloning vectors and host strains: nucleotide sequences of the M13mp18 and pUC19 vectors. Gene 33: 103-119.

3. Birnboim HC, Doly J (1979) A rapid alkaline extraction procedure for screening recombinant plasmid DNA. Nucleic Acids Res 7: 1513-1523.

4. Sambrook J, Russell DW (2001) Molecular Cloning: A Laboratory Manual: Cold Spring Harbor Laboratory Press.

5. Altschul SF, Madden TL, Schaffer AA, Zhang J, Zhang Z, et al. (1997) Gapped BLAST and PSI-BLAST: a new generation of protein database search programs. Nucleic Acids Res 25: 3389-3402.

6. Vallon O (2000) New sequence motifs in flavoproteins: evidence for common ancestry and tools to predict structure. Proteins 38: 95-114.

7. Iwaki H, Shimizu M, Tokuyama T, Hasegawa Y (1999) Purification and characterization of a novel cyclohexylamine oxidase from the cyclohexylamine-degrading *Brevibacterium* *oxydans* IH-35A. Journal of Bioscience and Bioengineering 88: 264-268.

8. Leonard PM, Smits SH, Sedelnikova SE, Brinkman AB, de Vos WM, et al. (2001) Crystal structure of the Lrp-like transcriptional regulator from the archaeon *Pyrococcus* *furiosus*. EMBO J 20: 990-997.

9. Platko JV, Calvo JM (1993) Mutations affecting the ability of *Escherichia* *coli* Lrp to bind DNA, activate transcription, or respond to leucine. J Bacteriol 175: 1110-1117.
